# Risk Assessment and Source Identification of Toxic Metals in the Agricultural Soil around a Pb/Zn Mining and Smelting Area in Southwest China

**DOI:** 10.3390/ijerph15091838

**Published:** 2018-08-25

**Authors:** Jinnan Wu, Jian Long, Lingfei Liu, Juan Li, Hongkai Liao, Mingjiang Zhang, Chang Zhao, Qiusheng Wu

**Affiliations:** 1 Guizhou Provincial Key Laboratory for Information System of Mountainous Areas and Protection of Ecological Environment, Guizhou Normal University, Guiyang 550001, China; wujinnan1214@foxmail.com (J.W.); liulfsuns@126.com (L.L.); liaohongkaii@163.com (H.L.); zhangmj33@163.com (M.Z.); change105@163.com (C.Z.); wqshengsf@gmail.com (Q.W.); 2 Department of Geography and Environmental Science, Guizhou Normal University, Guiyang 550001, China; lijuan_113@126.com

**Keywords:** Pb/Zn mining and smelting area, toxic metals, agricultural soil, spatial distribution, source identification, risk assessment

## Abstract

Mining and smelting activities are the primary sources of toxic metal pollution in China. The purpose of this study was to investigate the pollution risk and identify sources of metals in the arable soil of a Zn/Pb mining and smelting district located in Huize, in Southwest China. Topsoil (346) and profile (three) samples were collected and analyzed to determine the total concentrations of eight toxic elements (Cd, Hg, As, Pb, Cr, Cu, Zn and Ni). The results showed that the mean Cd, Hg, As, Pb, Cr, Cu, Zn and Ni concentrations were 9.07, 0.37, 25.0, 512, 88.7, 239, 1761 and 90.3 mg/kg, respectively, all of which exceeded both the Huize and Yunnan soil background levels. Overall the topsoil was quite acidic, with a mean pH of 5.51. The mean geoaccumulation index (*Igeo*) revealed that the pollution level was in the order of Pb > Zn > Cd > Hg > As > Ni > Cu > Cr. The ecological risk index (*E_i_*) indicated that there were serious contamination risks for Cd and Hg, high risk for Pb, moderate risk for As, and Cd and Hg were the dominant contributors to the high combined ecological risk index (*E_r_*) with a mean parameter of 699 meaning a serious ecological risk. The Nemerow pollution index (*P_n_*) showed that 99.1% of soil samples were highly polluted or worse. Horizontally, high concentrations of Cd, Hg, As, Pb and Zn appeared in the north and middle of the study area, while Cr, Cu and Ni showed an opposite trend. Vertically, as the depth increased, Cd, Hg, As, Pb and Zn contents declined, but Cr, Cu and Ni exhibited an increasing trend. The mobilities of the metals were in the order of Zn > Cd > Hg > As > Pb. Horizontal and vertical distribution, coupled with correlation analysis, PCA and CA suggested that Cd, Hg, As, Pb and Zn mainly came from the anthropogenic sources, whereas Cr and Ni had a lithogenic origin. The source of Cu was a combination of the presence of parent materials as well as human activities. This study provides a base for the local government to control the toxic metal pollution and restore the soil environment system and an effective method to identify the sources of the studied pollutants.

## 1. Introduction

Mining and smelting activities greatly promote employment and considerably benefit the domestic economy. Mineral extraction and its related industries, however, inevitably damage the soil’s natural texture and structure and bring about massive environmental toxic metal pollution into the neighborhood by emitting exhaust, discharging wastewater and acid mine drainage, open-piling and burying untreated slag and tailings during the industrial procedures [1,2,3,4]. As the industrial scale grows, the toxic metals accumulate gradually in soil and when the maximum limits the environment can tolerate are reached, they will exhibit strong ecotoxicity and bioaccumulation in the food chain due to their persistence and non-biodegradable properties. They can enter animals’ bodies via the soil-plant system and eventually pass into humans and harm people’s physical health and well-being [5,6,7].

For the last two decades, being the largest emerging economy in the world, China has witnessed a rapid growth creating much wealth in mining and non-ferrous metal smelting. Consequently, the issue of metal pollution has become more and more severe and it is urgent to tackle this tricky problem [8,9]. With the dramatical growth of industry and demand for non-agricultural usage, the amount of arable land in China had dropped to a dangerous level that can hardly supply future generations with enough food. Simultaneously, the quantity of farmland that has been polluted by metals has expanded rapidly so that the productivity of arable land in China is gradually declining [10]. The safety of agricultural soil is vital in modern society for food production, on which human safety largely depends [11]. Therefore, the arable land soil in the mining and smelting area can be more easily contaminated and its ecosystem is so vulnerable that many scholars around the world have paid more attention to this [12,13,14,15]. Besides, mining activities are not the only primary source of metals in polluting agricultural soil, but sewage irrigation [11], transportation emissions [16], disposal of household trash [17] and the excessive use of agrochemicals and manure [13] can result in massive arable land contamination. Therefore, to precisely identify the sources and pathways of the metals and accurately quantify the over-accumulations of metals is extremely essential for the purpose of being fully aware of the status quo of toxic metal pollution in local areas and providing useful basic knowledge for metal pollution treatment and remediation. 

Spatial distribution analysis combined with multivariance statistics methods including correlation analysis, principal component analysis (PCA) and cluster analysis (CA), are effective methods to determine the possible sources of metals [18]. Geographical information system (GIS) has been widely applied in the research fields of environmental monitoring, source identification and risk evaluation, the majority of which have been reported using plain visualization or bi-dimensional figures to display the results [4,12,19,20,21]. As geo-information technology has advanced, three-dimensional visualization and 3D GIS technology have matured and it is inevitable to expand current GIS applications into three-dimensional presentations with more realistic and intuitive features [22,23]. However, few studies have introduced tridimensional methods into the research field of toxic metal pollution in soil.

Huize County, in Yunnan Province, is a famous area worldwide for its abundance of non-ferrous metal ores, especially lead-zinc ore [24] and copper ore [25]. Zhehai, a town under Huize’s jurisdiction, has a long history of lead-zinc mining and a smelting industry dating back to the 1960s. Due to the long-term industrial activities, the production sites of enterprises have accumulated a great deal of smelting waste and drainage, becoming potential sources of pollution and posing serious threats to the ecological and public health. Authors such as Li et al. [26] and Qi et al. [27] have studied the metal concentration and pollution degree in the soil around Zhehai district, but their studies were unable to represent the overall status quo of pollution by the eight main toxic metals in Zhehai and the sources of these eight toxic elements haven’t been discussed before. Therefore, we selected the surface and soil profile of Zhehai arable soil as the research object, and used tridimensional distribution visualization and multivariate statistics, including correlation analysis, PCA and CA, to achieve the following aims: (i) to investigate the metals concentration in cultivated soil of Zhehai; (ii) to assess the geoaccumulation, pollution degree, potential ecological risk of metals; (iii) to determine the spatial distribution of metals both horizontally and vertically and identify the common sources of these metals. 

## 2. Materials and Methods

### 2.1. Study Area

**Figure 1:**
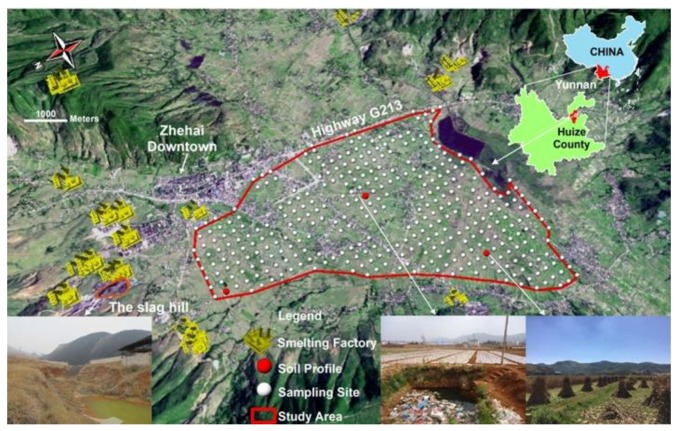
Map of the study area and location of the sampling sites in Zhehai, Yunnan Province, China.

The study area is located in Zhehai (103°34′ E–103°40′ E, 26°30′ N–26°33′ N) shown in Figure 1, a town in the northeastern part of Huize County in Yunnan Province, China. The Huize district is situated in the southwestern margin of the Yangtze Block, the southern part of the Northeastern Yunnan basin. It is the central part of the Sichuan-Yunnan-Guizhou triangular area of poly-metallic Pb/Zn mineralization. The geological background of the study area has ‘double-layer’ structure which consists of a weathered Permian basalt as the overlying cover rocks and dense massive Permian basalt as the lower basement [24,25]. The average elevation of the territory is around 2120 m, and the main soil type is Alfisols. The town is characterized by a humid subtropical climate, with an annual average temperature of 12.6 °C and annual average rainfall of 847 mm [26]. Maize and wheat are the main food corps for the residents. The slag hill shown at bottom left of Figure 1 is the biggest and most dangerous waste slag pile in town.

### 2.2. Sample Collection and Chemical Analysis

To fully investigate the metals accumulation in soils of Zhehai, the cultivated land in the south part of the town and left of the National Highway G213 were selected as the study area (Figure 1). A 200 m × 200 m fishnet was created using ArcGIS^®^ Desktop 10.3 (ESRI Inc., Redlands, CA, USA). The central point of each created grid was taken as the exact location of each sample. After deleting or moving the sampling sites which weren’t on the farmland soil, a total of 346 topsoil samples (0–20 cm depth) and three soil profiles (0–10 cm, 10–20 cm, 20–40 cm, 40–60 cm depth) were collected from the study area. Profile A was taken in the north near the smelters. Profile B was sampled in the center of the area adjacent to residential quarters. And Profile C was collected from the western corner (Figure 1). Hand-held GPS devices (G310, Unistrong, Beijing, China) were used to ensure that every sample was collected from the right spot as planned. Collected using stainless steel shovels and wooden scoops, every soil sample weighted about 1.5 kg and was a quarter of composite soil from five random thoroughly mixed subsamples collected within a 5 m radius around its sampling site. All the soil samples collected were stored in polyethylene bags, brought back to the lab, air-dried at room temperature for two weeks and passed through a nylon sieve with 2 mm aperture. Another portion of each soil sample was taken to sieve through a 0.15 mm-mesh nylon sieve for further chemical analysis. All the soil samples were analyzed for pH and 8 toxic elements (Cd, Hg, As, Pb, Cr, Cu, Zn, Ni). Soil pH was measured using a pH meter (PHS-3E, INESA, Shanghai, China) in a soil/water ratio of 1:2.5 suspension. A portion (0.2000 g) of each soil sample was digested with aqua regia for the determination of Hg and As, and a mixture of HNO_3_-HF-HClO_4_ for the measurement of Cd, Pb, Cr, Cu, Zn, Ni. The concentrations of Hg and As in soil were measured by atomic fluorescence spectroscopy (AFS-933, Jitian, Beijing, China). Cd, Pb, Cr, Cu, Zn aNi in soil samples were determined by flame-atomic absorption spectroscopy (ZEEnit700P, Analytik Jena, Jena, Germany). Reagent blanks, 10% duplicate samples and standard reference material (Certified Reference Materials for the Chemical Composition for Soil GSS 28, Chinese Academy of Geological Science, Beijing, China) were also included during the procedure to monitor the analytical accuracy and precision of the metal concentration analysis. The relative percentage difference of the duplicates was under ±5%, and the metal recoveries of elements in the reference material were within 86~107% (Table S1).

### 2.3. Assessment Methods of Metals Pollution

The contamination degree of metals in soils of the study area was evaluated using the geoaccumulation assessment (GAA) [28], potential ecological risk assessment (PER) [29] and Nemerow synthetic pollution estimation (NPI) [30,31].

#### 2.3.1. The Geoaccumulation Index (*Igeo*)

The *Igeo* index is widely utilized to evaluate metal pollution levels in soils and sediments [32,33], and is calculated according to Equation (1).

(1) $$Igeo=log_{2}\left(C_{i}/1.5\times B_{i}\right)$$

where *C_i_* represents the analyzed concentration of the metal *i* and *B_i_* is the concentration of Huize background value [34] of element *i*. The usage of the coefficient of 1.5 can make even small anthropogenic effect detectable [32]. The metals pollution level based on the obtained *Igeo* values were classified as follow [28]: (i) <0, unpolluted; (ii) 0–1, unpolluted to moderately polluted; (iii) 1–2, moderately polluted; (iv) 2–3, moderately to seriously polluted; (v) 3–4, seriously polluted; (vi) 4–5, seriously to extremely polluted; (vii) >5, extremely polluted.

#### 2.3.2. Potential Ecological Risk Index (*E_i_*)

The *E_i_* index, an indicator proposed by Hakanson [29], reveals the contamination risk of a single kind of metal to the environment, combines the toxicity of metals and the response of the environment with pollution level and risk assessments [17]. The synthesized potential ecological risk index (*E_r_*) can reveal the overall ecological hazard of all metals studied in soils or sediments. It is estimated as follows:

(2) $$E_{r}=\sum_{i=1}^{n}E_{i}=\sum_{i=1}^{n}T^{i}\times C^{i}=\sum_{i=1}^{n}T^{i}\times C_{i}/S_{i}$$

where *C^i^* represents the contamination factor of metal *i*, *C_i_* means the obtained concentration of metal *i* in soil samples, *S_i_* is the Huize background value [34] of element *i*, *T^i^* stands for the biological toxic factor of metal *i*. The relevant biological toxic factors for Cd, Hg, As, Pb, Cr, Cu, Zn and Ni are 30, 40, 10, 5, 2, 5, 1 and 5, respectively. The potential ecological risk level of the studied metals is classified based on the computed values of *E_i_* as follow [29]: (i) <40, low risk; (ii) 40–80, moderate risk; (iii) 80–160, high risk; (iv) 160–320, serious risk; (v) >320, severe risk. And the overall potential ecological risk degree according to *E_r_* is categorized as follow: (i) <150, low risk; (ii) 150–300, moderate risk; (iii) 300–600, high risk; (iv) 600–1200, serious risk; (v) >1200, severe risk.

#### 2.3.3. Nemerow Synthetic Pollution Index (*P_n_*)

The *P_n_* index is a kind of synthetic evaluation for the contamination degree of ground soils. The synthesis index can be computed as follows [31]:

(3) $$P_{n}=\sqrt{\left({\left(max\left\{C_{i}/S_{i}\right\}\right)}^{2}+{\left(\frac{1}{n}\sum_{i=1}^{n}C_{i}/S_{i}\right)}^{2}\right)/2}$$

where *C_i_* is the measured content of a certain element *i*, *S_i_* stands for the evaluation criterion of element *i*, which is usually referred to the scanning values of Chinese Soil Environmental Quality [35]. According to the pollution level, *P_n_* index can be categorized as follows [17]: (i) <1, unpolluted; (ii) 1–2, unpolluted to moderately polluted; (iii) 2–3, moderately polluted; (iv) 3–4, moderately to highly polluted; (v) 4–5, highly polluted; (vi) >5, very highly polluted.

### 2.4. Data Process and Statistics

Multivariate statistical analyses were performed using the open-source statistical software R, version 3.4.2 (R Project, Vienna, Austria) [36]. Pearson’s correlation analysis and Principal Component Analysis (PCA) with Varimax rotation were carried out to determine the relationship between the metals and to identify the possible origins of the metals [37,38,39]. Cluster Analysis (CA) with the standardized data using Ward’s method with Euclidean distance was performed to classify and cluster the metals and the soil samples which exhibited similarity [37,40]. Simple Kriging interpolation method was applied to estimate the spatial distribution of metals [41,42]. The location map of samples was conducted by ArcGIS^®^ Desktop 10.3 (ESRI Inc., Redlands, CA, USA). Three-dimensional spatial distribution maps and pollution degree heatmaps of metals were performed using Surfer^®^ 13 (Golden Software LLC., Golden, CO, USA).

## 3. Results and Discussion

### 3.1. Descriptive Statistics

The descriptive statistics of the metal concentrations and pH of the farmland topsoil are displayed in Table 1. The surface soils in the study area showed distinct accumulations of metals. The concentrations of eight metals (Cd, Hg, As, Pb, Cr, Cu, Zn, Ni) varied from 1.02 to 109, 0.04 to 3.36, 4.96 to 826, 73.4 to 10,059, 50.1 to 214, 59.4 to 359, 208 to 22,676, and 65.2 to 116 mg/kg, respectively. The mean Cd, Hg, As, Pb, Cr, Cu, Zn and Ni concentrations were 9.07, 0.37, 25.0, 512, 88.7, 239, 1761 and 90.3 mg/kg, respectively. The coefficient of variance (CV) values of metals decreased in the order: As (2.07) > Cd (1.45) > Pb (1.43) > Zn (1.18) > Hg (0.81) > Cr (0.27) > Cu (0.16) > Ni (0.11). The high CV values of As, Cd, Pb, Zn and Hg showed their high variability, indicating that contents of these metals greatly differed from one sampling site to another. The CV of Cr showed moderate variability, while Cu and Ni showed low variability. The large CV values of the metals concentrations in soils were seen as high geochemical variability [43]. Only the kurtosis value of Ni was lower than zero, while the kurtoses of the other elements were higher than zero, which indicated that the distributions of these metals except Ni were steeper than normal. The skewness values of all metals excluding Cu were greater than zero, showing that these seven metals were shewed towards lower concentration. pH varied from 4.14 to 7.34, and the majority were lower than 7, with the mean pH of 5.51, showing that the topsoil in the study area had an acidic pH surrounding. The data of pH and all toxic elements failed to pass the normality test, which suggested that anthropogenic activities brought much extrinsic input of acid and toxic metals into the topsoil [44].

The average concentrations of all eight metals in the study area were higher than the natural environment background values of Huize County, Yunnan Province and China [26,34], revealing that the topsoil of farmland in the study area was massively polluted by metals. The most severe one was Cd, which was 41 and 10 times larger than the background values of Yunnan and Huize, respectively.

The next metals were Pb, Zn, Hg, As and Ni with levels 17, 14, 6, 3 and 1 times higher than the background values of Huize, respectively. Cr and Cu were slightly over the Huize background. High metal concentrations combined with high CV values like Cd, Pb, Zn, As and Hg implied that anthropogenic activities played the main role in their accumulation [44]. The mean concentrations of the metals except Hg were over the CSQG [45]. Metals’ mean concentrations excluding As and Cr exceeded the DPL target values, whereas only the average concentrations of Cu and Zn were higher than the intervention values recommended in the DPL [46]. According to the related pH values of soil samples and scanning values of the CSEQ [35], the over-limit rate of Cd reached 100%, and Cu, Zn, Pb and Ni followed with the rate of 99.7, 99.4, 98.6 and 91.9%, and the over-limit rates of As, Hg and Cr were at very low levels with the percentages less than 7% for As and 2% for Hg and Cr instead. Obviously, the metals with more severe over-limit rates were Cd, Cu, Zn, Pb and Ni regarding to the CSQG [45] and target values of DPL [46]. According to the intervention values of the CSEQ [35], 95.7% and 46.0% of soil samples exceeded the maximum allowable values of Cd and Pb, respectively, which meant that the arable soil in the study area needed restoration intervention immediately and was not suitable for food production. Compared to intervention values of the DPL, Cu and Zn also reached high levels of pollution with the over-limit rates of 91.0% and 67.3%, respectively. On the other hand, the percentages of Hg, As, Cr and Ni concentrations exceeding intervention values remained low. The results in this study showed similarity with a former paper [26], which reported that the topsoil around the lead-zinc smelter in Zhehai, was acidic with an average pH of 6.90, and was greatly enriched with all toxic metals, with average concentrations (mg/kg) of Hg (0.60), As (29.9), Cr (104), Cu (239), and Ni (75), and more seriously contaminated with Pb (712), Cd (12.8) and Zn (1688). Likewise, the surface soils were also contaminated with Cd, Pb and Zn in another lead-zinc mining area in Zhaotong [47] and even more seriously in Lanping, in Yunnan Province [48]. Generally, the metals concentrations of farmland soil in Zhehai were higher compared with concentrations reported in soils from agricultural land [6,21] and other Pb-Zn mining areas in China [15,44,49] and in other parts of the world [4,50,51], which was likely to be due to the longer industrial history of mining and smelting lead-zinc ore.

### 3.2. Assessment of the Metals Contamination

#### 3.2.1. GAA and PER 

**Figure 2:**
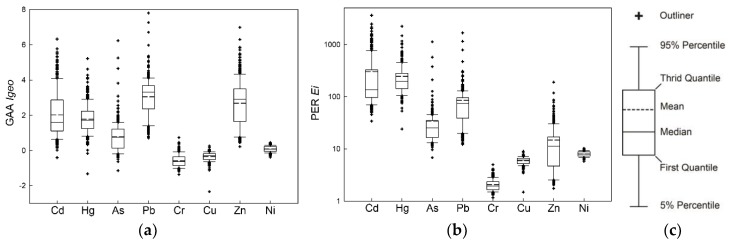
Boxplots of GAA (*Igeo*) (**a**) and PER (*E_i_*) (**b**), and the Legend (**c**) for metals in cropland soils of Zhehai. Notes: GAA: geoaccumulation assessment, PER: potential ecological risk assessment.

The obtained *Igeo* index of GAA in the cropland soils of the study area are shown in Figure 2. The average *Igeo* values were 2.02 (−0.40~6.33) for Cd, 1.78 (−1.32~5.22) for Hg, 0.78 (−1.14~6.24) for As, 3.05 (0.71~7.80) for Pb, −0.59 (−1.36~0.73) for Cr, −0.35 (−2.34~0.26) for Cu, 2.69 (0.22~6.99) for Zn, 0.08 (−0.38~0.45) for Ni, with the decreasing order of Pb > Zn > Cd > Hg > As > Ni > Cu > Cr. The mean *Igeo* of Pb pointed to a moderately to seriously polluted level, and Cd and Zn were at a moderately polluted degree. As and Ni revealed unpolluted to moderately polluted levels, while the indices of Cu and Cr suggested that the topsoil in Zhehai was generally unpolluted with these metals. The contamination reaching the seriously polluted level or worse accounted for 22.0%, 8.09%, 1.45%, 61.3%, 48.3% for Cd, Hg, As, Pb and Zn, respectively, but Cr, Cu and Ni could never be found at a seriously polluted level.

The *E_i_*s of metals displayed in Figure 2 ranged from 34.0 to 3628 (mean 302.32) for Cd, 24.0 to 2237 (mean 246) for Hg, 6.79 to 1131 (mean 34.3) for As, 12.2 to 1676 (mean 85.4) for Pb, 1.67 to 4.98 (mean 2.06) for Cr, 1.49 to 8.98 (mean 5.97) for Cu, 1.75 to 191 for (mean 14.8) Zn and 5.78 to 10.3 (mean 8.01) for Ni. which were reduced in the sequence of Cd > Hg > Pb > As > Zn > Ni > Cu > Cr. According to the mean values of *E_i_*, the topsoil was at serious risk of Cd and Hg, high risk of Pb, moderate risk of As, and low risk of Zn, Ni, Cu and Cr.

The *Igeo* index can exhibit the enrichment level of metals derived from both geogenic and anthropogenic sources [32,33]. According to the *Igeo* indexes we calculated, Pb, Zn and Cd had more severe pollution among all the toxic elements. Cu and Ni pollution levels were not as serious as the results that was obtained using CSEQ and DPL as references, because the background values of Cu and Ni in the study area were quite high. Based on the consideration of background and ecological toxicology of metals, Cd, Hg, Pb and As were the top four hazard pollutants that dominated the potential ecological risk. Likewise, the soil of other Pb/Zn mining areas in China was generally under the high ecological risk level for Cd, Hg, Pb and As [15,52].

#### 3.2.2. The Synthetic Assessment

The NPI results of showed that the average *P_n_* had a value of 22.7, indicating a very highly polluted level. The range of *P_n_* was from 2.54 to 260, 99.1% of which was higher than 4, indicating that the soil samples were highly polluted or worse. The synthetic index *E_r_* varied from 151 to 5354, with the mean of 699, revealing that average level of the study area was at serious ecological risk. The percentage of serious to severe risk was 38.4%, suggesting that extremely high values of *Ei* affected the overall risk degree being more severe. The *E_i_*s of element Cd and Hg were the dominant contribution to high *E_r_* parameter, averagely accounting for 36.3% and 39.4% of the total, respectively. These two synthetic parameters revealed that the arable soil in the study area was at a serious pollution level regarding to their means and maximums, which posed a serious hazard to the local people’s health and the ecosystem. As a mineral-abundant area, the southwest part of China, especially Yunnan, faces a difficult situation for the soil toxic metal pollution with high geoaccumulation and serious ecological risk in soil of the mining and smelting area [8,32]. Soil treatment and restoration actions must be taken.

### 3.3. Spatial Distribution of Metals, pH and Pollution Degree

**Figure 3:**
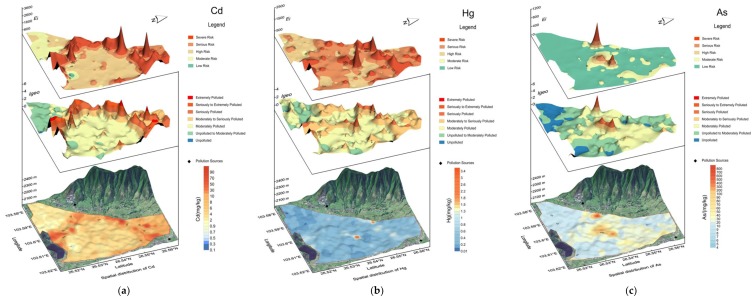

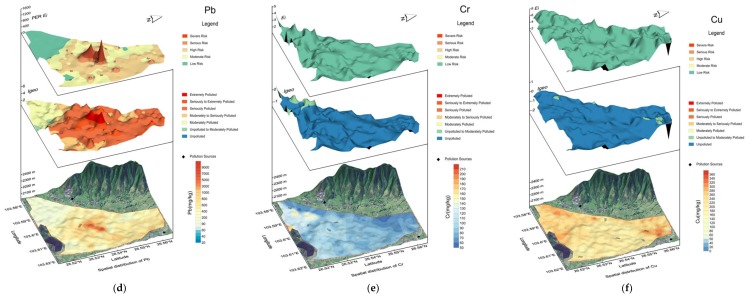

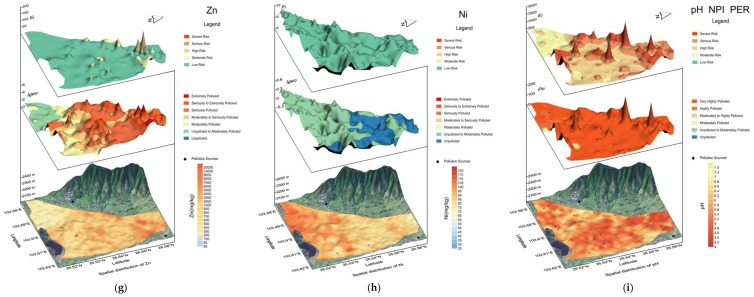
Spatial distribution maps of metal concentration and tridimensional heat maps of *Igeo* index and *E_i_* index of Cd (**a**), As (**b**), Hg (**c**), Pb (**d**), Cr (**e**), Cu (**f**), Zn (**g**), Ni (**h**) and spatial distribution maps of pH and tridimensional heat maps of *P_n_* and *E_r_* index (**i**).

The pollution hotspots and possible sources of metals can be identified effectively using spatial distribution as described in reference [37]. The spatial distribution patterns of metals in the study area are presented in Figure 3. The contents of metals are displayed by the color gradient from cold to warm color, which indicates metals vary from low to high concentrations. The scanning values of the CSEQ are taken as the baseline, and the colors of metals would turn red if their contents exceed the limits. Otherwise, their colors would remain blue. The distribution maps shown in Figure 3 demonstrated that the toxic metals were over the limits to a different extent. Cd, Pb, Cu, Zn and Ni were higher than the scanning values of the CSEQ within the whole area. Cr exceeded the limit merely in the western part, whereas the high content of Hg and As lay was generally in the middle. The soil pH map (Figure 3) indicated that the acidity of topsoil in the area was quite strong and widely distributed. 

Combined with the analyses above, the distribution of *Igeo* presented in Figure 3 indicates that Cd, Hg, As, Pb and Zn content accumulated in the soil to varying degrees because of the human activities, while Cr, Cu and Ni mainly derived from the parent materials according to their slight accumulation. Cd, Hg, As Pb and Zn are distributed approximately a similar trend that the high content region covered the most parts from the south to the north instead of the western corner, and extreme values appeared in the middle of the area. They probably had the same source [39], which was the direct emission of exhaust [17,53] and discharge of wastewater and residue from the lead-zinc smelting enterprises, and the surface runoff fed by acidic drainage and leaching liquid containing lots of metals [4,51]. The study area was located in a basin surrounded by mountains. So close was the terrain that exhaust fumes could barely scatter but held together in the valley or mostly move towards the north [26], a narrow exit, which made metals carried by fumes settle into the soil. Then streams and rainfall would wash out through the solid waste and polluted surface soil and carry a lot of metals to the central low areas, gathering and infiltrating into the subsoil and eventually contaminating the underground water [54]. Then the contaminated surface water and underground water became the main water sources for sewage irrigation which repeatedly polluted the soil [55]. Metals hotspots also appeared in the center, which adjoined the local residential quarters, suggesting that people’s daily activities such as improper disposal of household waste [38,56] and traffic exhaust [57] might aggravate the metals contamination. Although Cu, Cr and Ni contents went beyond the allowed environment limits [35], they scarcely contaminated the soil if the soil background is subtracted according to *Igeo*.

Besides, Cr and Ni showed a completely opposite distribution pattern where the high concentration was in the west and low concentration was in the most parts of the area which was totally different from Cd, Hg, As, Pb and Zn, indicating that they might originate from natural parent materials [39]. Although the lithogenic source dominated the concentration of Cu, an anthropogenic input also slightly contributed its contamination according to the mixed distribution pattern whereby a high content existed both in the north and west of the area. The distribution of *E_i_* for Cd and Hg showed that the high risk covered almost the whole area, while the high risk for As and Pb only appeared in the central part. And the ecological risk of Zn, Cr, Cu and Ni all maintained a quite low level. 

The synthetic assessment NPI shown in Figure 3 exhibited that very highly polluted regions could be found all over the area, and the spatial pattern was similar to that of Cd, which meant that Cd polluted the soil massively and contributed the most to the overall pollution degree. The synthetic PER index *E_r_* showed that the area was at high ecological risk or even higher risk except for its western part.

### 3.4. Vertical Distribution of Metals

**Figure 4:**
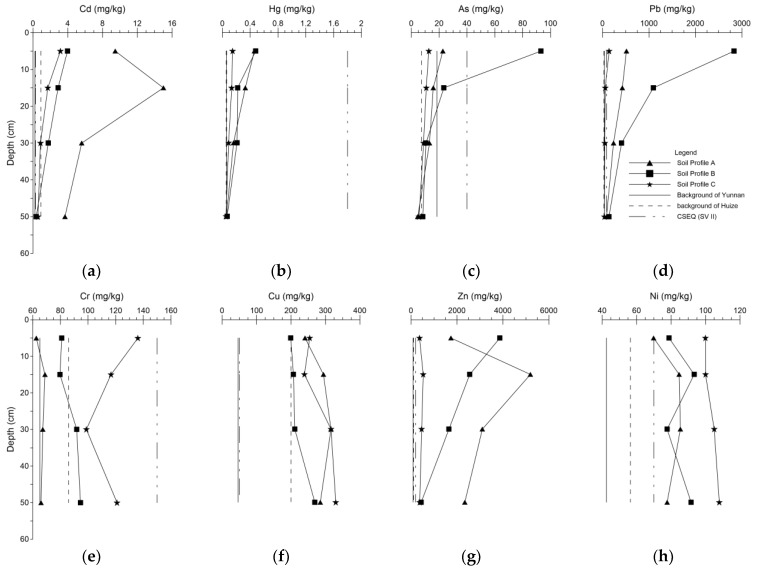
Vertical distribution of metal concentrations of Cd (**a**), Hg (**b**), As (**c**), Pb (**d**), Cr (**e**), Cu (**f**), Zn (**g**) and Ni (**h**) of Profile A, B and C.

The vertical distribution of metal contents in soil profiles is presented in Figure 4. Cd, Hg, As, Pb and Zn accumulated significantly in the 0–20 cm depth range of Profile A and B, while accumulation in Profile C in surface layer was negligible as compared to A and B. The results of the concentration variation as a function of depth showed that obviously different distribution patterns divided these elements into two groups. In general, the concentrations of Cd, Hg, As, Pb and Zn decreased drastically from topsoil to subsoil and accumulated greatly in the 0–20 cm depth range. While with the increasing depth, the concentrations of Cr, Cu and Ni remained largely unchanged or became slightly higher, which totally differed from the former metals. Based on the vertical difference, these two divided groups of metals probably originated from two different sources, which was consistent with the result of their spatial distributions. As focusing on the first group’s elements, Cd and Zn profiles presented a similar trend that mass accumulation had transferred into subsoils in 10–20 cm depth range in Profile A, revealing that the downward mobility of Cd and Zn was very strong under these acidic and seriously polluted conditions. Unlike them, As, Pb and Hg abruptly reduced from topsoil at 0–10 cm to subsoil in the 10–20 cm depth range. Li et al. [52] also observed a consistent result that Cd and Zn contents increased from the top of 0–10 cm to the depth range of 10–20 cm first and then decreased significantly around a Pb/Zn smelter in Hunan, China. According to the previous studies in the same area [26,27] and other Pb/Zn smelting area [51,52,58], Cd, Hg, As, Pb and Zn exhibited the same accumulation in 0–20 cm depth range and reduction in deeper depth as this study. Li et al. [26] revealed that the concentration of Ni increased as depth going deeper in profiles around Zhehai Pb/Zn smelter, and Ghayoraneh and Qishlaqi [51] also reported that Cr, Cu and Ni contents showed an increasing trend with depth in soil profiles near an Iranian Pb/Zn smelter, which was similar to our results.

The factors that would influence their mobilities mainly include the soil physiochemical properties [59], metal fraction properties [26] and surface-deposited concentration [60]. Acidic soil pH conditions could enhance metal migration capability [61,62]. Metal concentrations in the surface horizon also positively affect their leaching ability [63]. According to the descending rates from the soil surface to subsurface layer of Cd, Hg, As, Pb and Zn in this study, the vertical migration ability decreased in the order of Zn > Cd > Hg > As > Pb. The previous study produced a similar result in the same area [26] of the metals mobilities. Our result of Zn migration ability was stronger as compared to other Pb/Zn smelting area [58,64], which might result from the stronger acidic soil pH condition and higher content in the surface deposit. Li et al. [26] drew the conclusion that higher proportions of Cd and Zn in the acid-soluble fraction might lead to deeper downward migration than that of Pb, and with the increase of total Zn content, the proportion of Zn in non-residual form would increase, and so would the downward migration ability of Zn. Metal fractionation forms would also influence metal mobility significantly [27,51,59,64,65], but this present study did not cover this part. In the future research, the subject would be well analyzed and specifically discussed.

Profile A was mainly influenced by the industrial smelting activities, which might be explained by the high mobility of Zn and Cd in the strong acidic and high concentration conditions in the surface soil. With high concentrations of Hg, As and Pb in surface soil, not only the exhaust and residue from the smelters but more importantly, the household waste and vehicle fumes local people created would contaminate Profile B considering its residential-adjoined location. Profile C might represent what a barely polluted soil profile might be in Zhehai.

### 3.5. Correlations of Metals and pH

Pearson correlation analysis was conducted to reveal the interrelationship between the metals so as to preliminarily determine their common origin sources [6,17,37,57]. The raw data of Cd, Hg, As, Pb, Cr and Zn concentrations were subjected to a logarithmic transformation to create an approximatively normal distribution to reduce the potential influences of outliers [38]. The computed results are shown in Table S2. There existed significantly positive correlations between Cd, Hg, As, Pb and Zn, especially between Zn and Pb, Cd, As, Pb, As with the 0.0001 significance level (r from 0.68 to 0.83), which meant that they probably stemmed from the same source. Ni and Cr were significantly positively correlated with each other, but they both had significantly negative correlations with Cd, Hg, As, Pb and Zn, which indicated that the origin of Cr and Ni was likely to differ from the other elements. A mixed interrelationship appeared that Cu displayed significantly positive correlations with Cd, Hg and Ni, while negative correlations existed between Cu and Pb, Cr. This might suggest Cu had a combined source. Similar results could be seen in other papers [30,41] Weak correlations could be found between pH and the metals except for the Cd, Cr and Ni.

### 3.6. Source Identification Using PCA and CA

As the extension of the correlation analysis, PCA was carried out to distinguish the possible origins of metals [11,30,39,53,66]. The PCA result was shown in Table S3. Three principal components (Factors) with eigenvalues above 1 were extracted, whose cumulative proportion accounted for 84.0% of the total variance. The first principle component (Factor 1) with a variance loading of 52.8%, mainly included Cd, Hg, As, Pb and Zn, with the higher variance of 0.70, 0.82, 0.87, 0.86 and 0.83, respectively. Meanwhile, these five metals were significantly correlated with each other, suggesting that they originated from human activities such as large amounts of emission and improper disposal of wastes from the lead-zinc smelting industry or household garbage and fossil-fuel consuming vehicles. There have been numerous papers ensuring the result of homogeneous origin of Cd, Hg, Pb, As and Zn classified together by PCA in agreement with our study [39,40,67]. Factor 2, accounting for 17.9% of the total variance, was dominated by Cr and Ni, with a variance of 0.90 and 0.83, respectively. According to the high background values, they were probably derived from soil parent materials, which was consistent with previous papers [20,68,69]. Cu was the only element in Factor 3 as shown in Table S3, with a variance of 0.93, which revealed that Cu had a mixed source including both anthropogenic and lithogenic source. Davis et al. [18] also used PCA to confirm that Cr and Ni mainly come from natural source, and Cu had a combined source from both human and natural activities.

**Figure 5:**
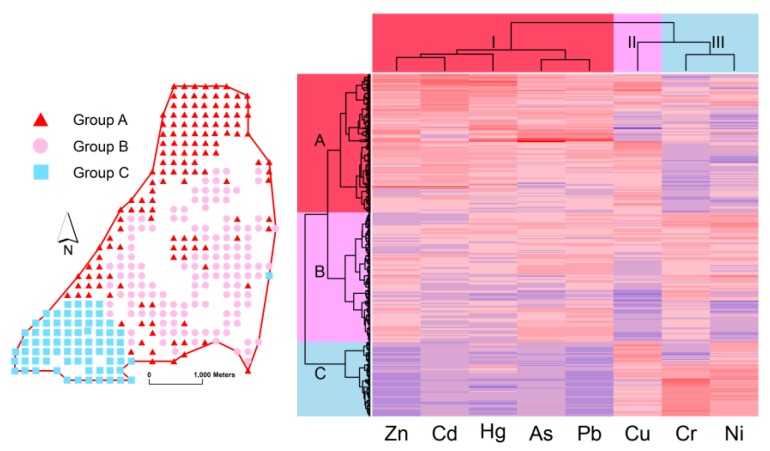
Dendrogram results of hierarchical cluster analysis for metals and soil samples. Grids in a row with blue-to-red color gradient represents the concentration of metals varied from low to high in a topsoil sample.

As a method used for confirmation of PCA results [5,18], the results of hierarchical CA for toxic elements and soil samples was illustrated in a plane heatmap in Figure 5. The clustering tree on the X-axis above was the CA result for metals. On the Y-axis left was the dendrogram for surface soil samples. Each thin grid represented with blue-to-red color gradient indicates the concentration varied from low to high of one metal in a topsoil sample. The classification of metals obtained by CA was consistent with the results of PCA. They were divided into three groups, I, II and III. Members of Group I, including Zn, Cd, Hg, As and Pb, came from the anthropogenic origin. Group III containing Cr and Ni originated from pedogenic factor. And Cu was the only element in Group II stuck between Group I and II, indicating that it was affected by double groups. The statistical results above were further justified by many studies [11,14,17,30,53]. The findings obtained by correlation analysis, PCA and CA were solid for the reasons that Zn, Pb, Cd, As and Cu in the lead/zinc ore and copper ore [24,25] would be released by emitting exhaust, discharging wastewater and acid mine drainage. Hg, Cd and As carried by the exhaust would return to soil through atmospheric deposition. Combusting coal and other fuels during the smelting procedure can also produce Pb, Cd, As and Hg [17,26]. Also, household trash, gasoline-fuel vehicle using [57] and chemical fertilizer and pesticides application [11] could bring Pb, Zn, Cd, As Cu and Hg in soil. On the other hand, Cu, Cr and Ni usually appears in the bedrock and soil mineral [17,18,41].

The 346 samples were also classified into three groups displayed in the Y-axis, Group A, B and C. Group A included 140 samples, whose color mostly were deep red for Zn, Cd, Hg, As, Pb and Cu, and light blue for Cr and Ni, with an average content (mg/kg) of 2987, 17.2, 0.55, 36.8, 716, 253, 73.5 and 84.9 respectively, which exceeded scanning values of CSEQ [35] and CSQG [45] greatly. In Group A, especially, Zn, Cd, Pb, and Cu were even above Dutch intervention limit [46]. Most of the Group A members were gathered in the north of the study area, and there were some points scattering in the middle and left edge, suggesting that they kept away from the local resident but close to Pb/Zn smelters, and their main source of pollution was from direct emission of exhaust and discharge of wastewater and residue from the lead-zinc smelter. Group B including 131 samples was mainly located in the center of the area in the neighborhood of the residential places, with lower mean concentrations (mg/kg) of 1275, 4.25, 0.28, 20.9, 515 and 212 for Zn, Cd, Hg, As, Pb, Cu, and a little higher mean (mg/kg) of 91.5 and 89.9 for Cr and Ni, indicating that this region was affected not only by the Pb/Zn industry, but by household and transportation pollution and agricultural activities occurring as part of the daily routines of the local people, such as improper disposal of garbage and wastewater, vehicle emission, and chemical pesticides and fertilizer usage. Group C contained 75 samples, whose Zn, Cd, Hg, As and Pb concentrations were the lowest as compared with Group A and B, while Cu, Cr and Ni were higher than others. These sampling sites lay together in the west corner on the left, revealing a concentration level of toxic metals in restrictedly polluted soil.

## 4. Conclusions

This study investigated the metals concentrations of cropland soil in Zhehai, a typical Pb/Zn mining and smelting area located in Yunnan Province, Southwestern China. Combined with spatial analysis, the pollution sources of the studied metals were identified and the results were further examined for consistency using correlation analysis, PCA and CA. The methods that this study applied combined multivariate statistics with both horizontal and vertical spatial distribution analysis, which could be a practical tool to fully analyze the possible origins of pollutants. Tridimensional visualization for the pollutants in topsoil was also adopted to demonstrate their horizontal distribution more closely to reality. Compared with the soil background values of Huize County, the metals concentrations of soil in the study area have accumulated to various levels with an acidic soil pH condition. The most severe ones were Pb, Zn, Cd, Hg, As, whose mean concentrations were 17.1, 14.8, 10.1, 6.2, 3.4 times higher than the Huize background values [34], while Cr, Cu and Ni displayed a lower contamination degree. Based on the scanning values of CSEQ [35], the rates of excess Cd, Cu, Zn Pb and Ni all accounted for 100, 99.7, 99.4, 98.6 and 91.9% of the total. The results of GAA indicated that the contents of Pb, Cd and Zn reached a dangerous level, and PER showed that the ecological risk declined from serious to low one in the order of Cd > Hg > Pb > As > Zn > Ni > Cu > Cr. 

Horizontally, high concentrations of Cd, Hg, As, Pb and Zn similarly appeared in the north and middle of the area. On the contrary, Cr and Ni exhibited an opposite trend, with a low content in the middle and north but high in the west corner, while Cu combined both spatial distribution patterns. Vertically, the concentrations of Cd, Hg, As, Pb and Zn decreased considerably from topsoil to deeper soil layer, while Cr, Cu and Ni concentrations changed slightly higher or remain constant all the way down, suggesting that these two groups of metals had non-homogeneous sources. The metal mobilities of the former group members declined in the sequence Zn > Cd > Hg > As > Pb. Correlation analysis, PCA and CA revealed that Cd, Hg, As, Pb and Zn were classified into one group and originated from anthropogenic sources, while Cr and Ni were categorized into another group, which mainly came from lithogenic sources. Cu was isolated being the only component of the third group, meaning that Cu pollution stemmed from both natural and man-made factors. All samples sites were divided into three groups. Group A was in the north of the area close to the Pb/Zn smelters, whose contents of Zn, Cd, Hg, As, Pb were the highest, indicating that smelting activities dominated their sources. Group B was in the middle near the residential area, which meant people’s daily activities also had a great influence. In the west corner was Group C, representing the natural or scarcely polluted soil should be in Zhehai. 

Therefore, the authorities should take the toxic metals’ both anthropogenic and lithogenic sources into account. For one thing, small and outdated smelting factories need shutting down and metals contents of the exhaust and wastewater of the smelting industry must meet the environment-allowed standards. For another, household trash must be well treated and vehicle exhaust must be rigidly restricted. The results concluded by this research can provide a basis of metal treatment and remediation for the local authority and an integrated method which combines spatial and statistical analyses is put forward by this study to identify the pathway of the studied pollutants.

## Figures and Tables

**Table 1:** Descriptive statistics of metals concentrations (mg/kg) and pH of topsoil in Zhehai.

Descriptive Statistics	Cd	Hg	As	Pb	Cr	Cu	Zn	Ni	pH
Mean	9.07	0.37	25.0	512	88.7	239	1760	90.3	5.51
Minimum	1.02	0.04	4.96	73.4	50.1	59.4	208	65.2	4.17
Median	4.08	0.30	18.4	448	83.1	244	1345	89.5	5.39
Maximum	109	3.36	826	10,058	214	359	22,676	116	7.34
SD	13.1	0.30	51.7	730	23.7	37.9	2073	10.4	0.69
CV	1.45	0.81	2.07	1.43	0.27	0.16	1.18	0.11	0.13
Kurtosis	22.1	35.2	178	104	2.89	0.76	34.9	−0.91	−0.57
Skewness	4.07	4.70	12.5	9.18	1.39	−0.33	4.65	0.07	0.41
BV of China ^a^	0.10	0.07	11.2	26.0	61.0	22.6	74.2	26.9	-
BV of Yunnan ^b^	0.22	0.06	18.4	40.6	65.2	46.3	89.7	42.5	-
BV of Huize ^c^	0.90	0.06	7.30	30.0	86.0	200	119	56.4	-
CSEQ (SV I) ^d^	0.30	1.30	40.0	70.0	150	50.0	200	60.0	<5.5
CSEQ (SV II) ^d^	0.30	1.80	40.0	90.0	150	50.0	200	70.0	5.5~6.5
CSEQ (SV III) ^d^	0.30	2.40	30.0	120	200	100	250	100	6.5~7.5
CSEQ (IV I) ^e^	1.00	2.00	200	400	800	-	-	-	<5.5
CSEQ (IV II) ^e^	2.00	2.50	150	500	850	-	-	-	5.5~6.5
CSEQ (IV III) ^e^	3.00	3.00	120	700	1000	-	-	-	6.5~7.5
CSQG ^f^	1.40	6.60	12.0	70.0	64.0	63.0	200	45.0	-
DPL(TV) ^g^	0.80	0.30	29.0	85.0	100	36.0	140	35.0	-
DPL(IV) ^h^	12.0	10.0	55.0	530	380	190	720	210	-
% Over CSEQ(SV)	100	1.16	6.65	98.6	1.16	99.7	99.4	91.9	-
% Over CSEQ(IV)	95.7	0.29	0.87	46.0	0	-	-	-	-
% Over CSQG	99.4	0	75.1	100	90.5	99.7	100	100	-
% Over DPL(TV)	100	48.6	16.8	98.8	26.0	100	100	100	-
% Over DPL(IV)	18.8	0	2.90	35.6	0	91.0	67.3	0	-

^a^ Background value of China, ^b^ Background value of Yunnan, ^c^ Background value of Huize [26,34]; ^d^ Chinese Soil Environmental Quality (Screening Values), ^e^ Chinese Soil Environmental Quality (Intervention Values) [35]; ^f^ Canadian Soil Quality Guidelines [45]; ^g^ Dutch Pollutant Limit (Target Value), ^h^ Dutch Pollutant Limit (Intervention Value) [46].

## Supplementary Materials

The following are available online at http://www.mdpi.com/1660-4601/15/9/1838/s1, Table S1: Measured and certified concentration (mg/kg) and metal recovery of reference materials. Table S2: Pearson correlation coefficients between the toxic metals and pH. Table S3: Factor loadings of the PCA results of the toxic metals in the study area.
