# Supplementary material for: Risk Assessment and Source Identification of Toxic Metals in the Agricultural Soil around a Pb/Zn Mining and Smelting Area in Southwest China
Source: Int J Environ Res Public Health. 2018 Aug 25;15(9):1838. doi: 10.3390/ijerph15091838 (PMC6165396; doi:10.3390/ijerph15091838)
Supplement: Supplementary file 1 [file ijerph-15-01838-s001.pdf]

Table S1. Measured and certified concentration (mg/kg) and metal recovery of reference materials.

	Cd	Hg	As	Pb	Cr	Cu	Zn	Ni
Measured Concentration of Reference Material	0.49~0.53	0.12~0.15	29.1~29.9	60.1~63.4	96.7~98.8	36.0~37.6	134~142	41.3~43.7
Certified Concentration of Reference Material [a]	0.52	0.14	28.5	61.0	94.0	38.0	134	43.0
Metal Recovery (%)	94~102	86~107	102~105	99~104	103~105	95~99	100~106	96~101

[a] Certified Reference Materials for the Chemical Composition for Soil GSS28, Chinese Academy of Geological Science.

Table S2. Pearson correlation coefficients between the toxic metals and pH.

	Cd	Hg	As	Pb	Cr	Cu	Zn	Ni	pH
Cd	1	0.68 ***	0.45 ***	0.46 ***	−0.43 ***	0.27 ***	0.76 ***	−0.30 ***	0.23 ***
Hg		1	0.63 ***	0.61 ***	−0.36 ***	0.16 **	0.62 ***	−0.29 ***	0.09 *
As			1	0.83 ***	−0.26 ***	−0.08	0.68 ***	−0.41 ***	0.06
Pb				1	−0.35 ***	−0.24 ***	0.79 ***	−0.51 ***	−0.05
Cr					1	−0.20 ***	−0.49 ***	0.64 ***	0.19 ***
Cu						1	−0.05	0.31 ***	−0.01
Zn							1	−0.51 ***	0.04
Ni								1	0.20 ***
pH									1

*** significant at the 0.0001 probability level, ** significant at the 0.01 probability level, * significant at the 0.05 probability level.

Table S3. Factor loadings of the PCA results of the toxic metals in the study area.

Toxic Metals	Variance			Communalities
	Factor 1	Factor 2	Factor 3	
Cd	0.70	−0.29	0.45	0.77
Hg	0.82	−0.13	0.27	0.76
As	0.87	−0.10	−0.17	0.80
Pb	0.86	−0.24	−0.30	0.88
Zn	0.83	−0.39	0.01	0.84
Cr	−0.20	0.90	−0.25	0.90
Ni	−0.29	0.83	0.34	0.89
Cu	−0.03	0.03	0.93	0.87
Cumulative loading (%)	52.8	70.7	84.0	-
